# *L. reuteri* in Supportive Periodontal Therapy—Are There Already Clinical Effects after 3 Months with One Lozenge a Day? A Double-Blind Randomized Placebo-Controlled Study

**DOI:** 10.3390/microorganisms12040648

**Published:** 2024-03-25

**Authors:** René Thierbach, Andreas Eigenmann, Jusef Naim, Matthias Hannig, Stefan Rupf, Madline P. Gund

**Affiliations:** 1 Medical Supply Center Ulm, German Armed Forces, 89081 Ulm, Germany; renethierbach@bundeswehr.org; 2 Private Practice Dr. Badke, 13055 Berlin, Germany; info@familienpraxis.berlin; 3 Clinic of Operative Dentistry, Periodontology and Preventive Dentistry, Saarland University, 66421 Homburg, Germany; naimjusef@gmail.com (J.N.); matthias.hannig@uks.eu (M.H.); stefan.rupf@uks.eu (S.R.); 4 Synoptic Dentistry, Saarland University, 66421 Homburg, Germany

**Keywords:** supportive periodontal therapy, supportive periodontal care, *L. reuteri*, probiotics, scaling and root planing, periodontitis

## Abstract

Aim: The purpose of this study was to investigate whether a clinical effect of *Lactobacillus reuteri* in supportive periodontal therapy can already be detected with a minimum dose of one tablet a day and a minimum observation and intake period of 3 months. Materials and Methods: 28 patients with stage III and IV periodontitis undergoing periodontal follow-up were randomly divided into two groups receiving a lozenge containing *L. reuteri* or a placebo preparation daily for 90 days. After 0, 4, 8, and 12 weeks, the parameters bleeding on probing (BoP), plaque control record (PCR), periodontal probing depth (PPD), and clinical attachment level (CAL) were recorded in the test and control groups. Results: The results sed a different effect of *L. reuteri* on the respective patients. In certain patients, clinical parameters worsened or remained largely unchanged. However, in other patients, there were positive effects on the clinical parameters. In the overall analysis, BoP was the only clinical parameter that was statistically significantly reduced. Conclusions: The oral administration of one lozenge per day for 3 months with *L. reuteri* in supportive periodontal therapy might have a positive influence on clinical parameters in supportive periodontal therapy, depending on the individual.

## 1. Introduction

The effects of *Lactobacillus reuteri* and probiotics in periodontal therapy are currently controversial. A 2017 review describes the limited, short-term effects of the probiotic as an adjunct to scaling and root planing, while pointing to studies with insufficient patient numbers and observation periods [1]. *L. reuteri* may be useful as an adjunct in initial periodontal therapy, while simultaneously emphasizing that the optimal dose still has to be determined [2]. Another review from the year 2020 describes that there is little evidence for probiotics to reduce inflammatory parameters in gingivitis. They again discuss heterogeneity and limited data as limitations [3]. Ramos et al. investigated the effect of systemic antibiotic and probiotic therapies (*L. reuteri*) as an adjunct to subgingival treatment, emphasizing that none achieved any additional benefits [4]. In their systematic review, Silva et al. studied the effect of adjunct probiotics on peri-implant treatment, complaining about insufficient evidence to confirm a beneficial effect [5]. Villafuerte et al. emphasize the pathogen-reducing effects of *L. reuteri* as an adjunct in periodontal treatment, while at the same time requesting high-quality randomized controlled trials to examine only one bacterial strain at a time [6]. In their scoping review, Routier et al. outline the place of probiotics in future periodontal treatment when treatment is individualized [7]. Jansen et al. also emphasize this approach [8]. Due to heterogeneity and different assessments of the results, as just shown in the EFP S3 level clinical practice guideline (treatment of stage I-III periodontitis), probiotics are currently not recommended as adjuncts to subgingival instrumentation [9]. The effect of *L. reuteri* in supportive periodontal therapy has hardly been investigated to date. In this context, it is particularly interesting to know whether patients have to take the lozenges for several months in order to see any effect at all in periodontally stable conditions or whether clinical improvements are visible even after a short time and minimal dosage.

The present study aimed to analyze whether a clinical effect of *Lactobacillus reuteri* could already be detected with a minimum dose of one tablet per day over a 3-month observation and intake period in supportive periodontal therapy.

## 2. Materials and Methods

The ethics committee of the Saarland Medical Association approved this double-blind, placebo-controlled study (No. 251/16). Patients who had received periodontal therapy and were in a periodontal aftercare program were included in the study. All patients were treated in the same private practice (Private Practice Dr. Badke and Partner, Berlin). The following aspects were defined as inclusion criteria: (1) healthy male or female patients with a minimum age of 18 years, (2) periodontal therapy with the diagnosis: generalized periodontitis stage III/IV. The exclusion criteria were the following: (1) antibiotic therapy within the last 6 months prior to study entry, (2) use of antibiotic medication during the course of the study, (3) use and regular use of an antibacterial mouth rinse, (4) smokers or former smokers who had quit smoking in the last 12 months, (5) pregnancy and lactation, (6) necrotizing ulcerative periodontitis, (7) acute intraoral injuries, (8) systemic diseases such as diabetes, rheumatism, liver and kidney diseases, neurological diseases, and immunological diseases, and (9) intake of medications that affect the periodontal tissue (phenytoin, ciclosporin, and nifedepine).

Patients who met all inclusion and exclusion criteria were eligible for participation in the study. Participation in the study was explained in detail. The method of implementation and intake of the study medication were communicated. Furthermore, potential risks were explained. After a detailed discussion, written informed consent was obtained from all study participants. The study design was not changed after approval by the ethics committee.

The size of patient groups was determined according to Cohen [10]. The primary endpoint was defined as the change in bleeding on probing. A significance level of 5% and a discriminatory power of 90% could be achieved in this study with a group size of 12 patients. This means a total of 24 patients for both study groups. In order to ensure the minimum number of 12 patients over the entire study period, more than 24 patients were included in the study. At the start of the study, there were 18 patients in each group (36 patients in total).

### 2.1. Experimental Design and Treatment Protocol

All participants in the study had previously undergone periodontal therapy at the private practice of Dr. Badke and Partners, Berlin. The periodontal therapy was performed at least 6 months before participating in the study and consisted of a full-mouth, one-stage disinfection approach [11] and a detailed explanation of the disease and oral hygiene instructions. The subgingival debridement (SD) was performed during a single appointment using an ultrasonic scaler (KaVo SONICflex, Biberach, Germany) under 0.12% chlorhexidine (CHX) irrigation and using hand instruments (curettes). All mucosal surfaces were disinfected with CHX 0.12% on a swab. Three months after SD, the periodontal parameters were re-examined. Furthermore, the patients were included in a periodontal follow-up program. All study participants were included in a 3-month recall program based on their periodontal diagnosis, according to Lang and Tonetti [12]. The baseline examination for the study consisted of a full-mouth measurement, at six sites, of the periodontal pocket depth (PPD), gingival recession (REC), bleeding on probing (BoP) and the full-mouth plaque control record (PCR) according to O’Leary (1972) [13]. The clinical attachment level (CAL) was calculated as the sum of the recession (REC) and probing depths (PPD). All examinations were performed by the same examiner (AE) using a UNC-15 periodontal probe (Hu-Friedy, Chicago, IL, USA). After the initial periodontal therapy, all patients received supportive periodontal care (SPC), including oral hygiene instructions and a full-mouth supragingival plaque removal using an ultrasonic scaler (KaVo SONICflex, Biberach, Germany) and hand instruments. All clinical manipulations and measurements were performed by one dentist (AE).

**Figure 1:**
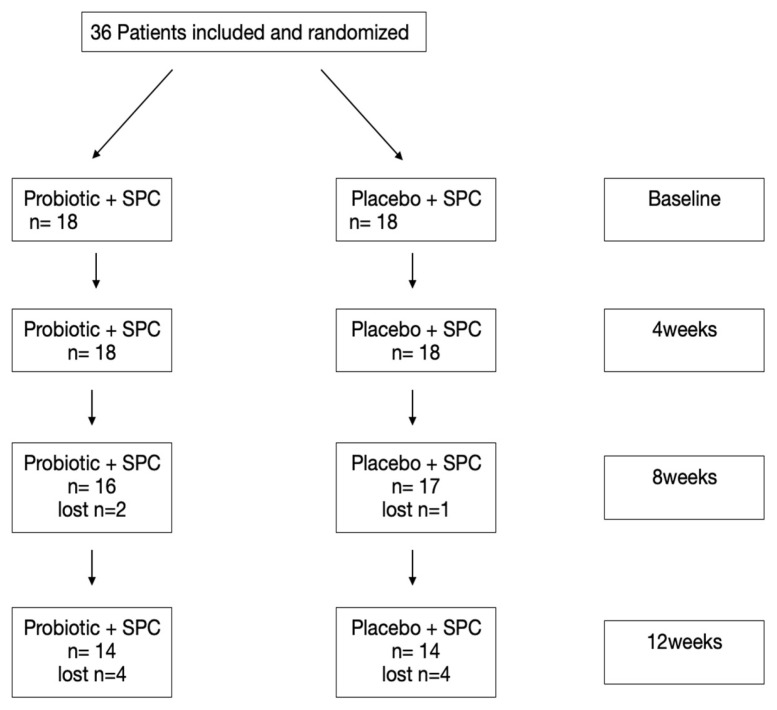
Study procedure. SPC = Supportive Periodontal Care.

The study coordinator (RT) randomly divided the study participants into two different groups: one group received the probiotic and the supportive periodontal care (test group, probiotic + SPC), the second group received the placebo and the supportive periodontal care (control group, placebo + SPC). The probiotic and the placebo were taken in the form of lozenges. In both groups, the probiotic was taken over a period of 12 weeks. One lozenge was taken daily. Follow-up examinations were carried out 4, 8, and 12 weeks after the first SPC treatment. At these appointments, a clinical evaluation of all baseline parameters was performed (BoP, PCR, PPD, and REC) (Figure 1).

### 2.2. Randomization

Patients who met all the criteria for participation in the study and consented to the study were randomly assigned. Specially labeled containers were handed out by the study coordinator (RT) to the investigator (AE). These were given to all study participants during the first SPC treatment, with corresponding instructions on how to take the medication. Further handovers of the study medication took place after 4 and 8 weeks. Membership in a particular study group (test or control) was not apparent to either the patients or the study staff. Only the study coordinator was able to recognize the affiliation with the different study groups. The study groups were unblinded after the end of the study period and the evaluation of the data.

### 2.3. Product under Investigation

The probiotic lozenges consisted of *L. reuteri* (1 × 10^8^ CFU) for each of the DSM17938 and ATCC PTA5289 strains (PerioBalance; BioGaia, Lund, Sweden). Both the probiotic and placebo lozenges were indistinguishable in shape, texture, and taste. Patients in each study group took the lozenges in the morning after brushing their teeth. Patients were asked not to take any other probiotic supplements during the study period.

### 2.4. Outcome Variables

Bleeding on probing (BoP) was defined as the primary parameter to be observed. PCR, PPD, and CAL were selected as additional secondary outcome variables. Clinical attachment level (CAL) was calculated as the sum of PPD and REC.

### 2.5. Examiner Calibration

The calibration of the clinical examiner (AE) was performed by determining the intra-examiner variability. For this purpose, the periodontal parameters PPD and CAL were determined in 10 periodontitis patients who were not part of the study population. At least 6 teeth per patient were measured in one quadrant. After 60 min, another measurement was performed on the same teeth. The deviations between the two measurements were then determined. The intra-examiner variability for PPD and CAL measurements was assessed and set at 0.18 mm for PPD and 0.21 mm for CAL.

### 2.6. Compliance and Adverse Events

To check whether the patients had taken the study medication, the patients were asked to bring the empty medication containers to the examination. Patients returned the containers with the probiotic or placebo lozenges at the 4, 8, and 12-week appointments. At the 0, 4, and 8-week appointments, patients received a new container with the study medication. The container always contained exactly as many lozenges as the patients needed until the next appointment. The examination appointments were scheduled accordingly. At each follow-up appointment, the study participants were asked about general health changes, any additional medication taken, and any adverse events (e.g., gastrointestinal complaints) that the patient had noticed.

### 2.7. Statistical Analysis

The data were analyzed using SPSS Statistics (SPSS Statistics Version 28, IBM, Chicago, IL, USA). The range, mean, and standard deviations were calculated for numerical values. The differences between the two means were tested using the Mann–Whitney test (Z). The Friedman chi-squared test (X2) was used to compare the observation groups measured during follow-up and, if found to be significant, a pairwise comparison was performed using the Mann–Whitney test (Z). Data were considered statistically significant if *p* < 0.05.

## 3. Results

The demographic data of the study patients are shown in Table 1. No significant differences (*p* > 0.05) were observed between the two groups. All clinical examinations were performed between June 2021 and April 2022. 36 patients were enrolled in the study, 28 were able to complete the study, four had to interrupt the study due to COVID-19 infection and were therefore unable to comply with the study protocol. Two patients moved away and two others had to take antibiotics due to illness and therefore no longer met the study inclusion criteria. No protocol compliance issues were noted in the 28 patients completing the study, and no adverse effects of the lozenges were mentioned by patients or observed by the investigator.

### 3.1. Bleeding on Probing (BoP)

**Figure 2:**
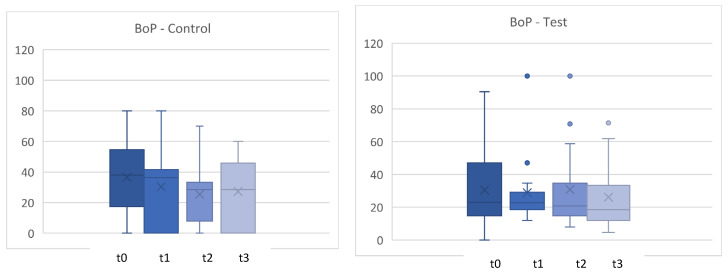
Bleeding on probing (BoP) in % for t0, t1 (4 weeks), t2 (8 weeks), t3 (12 weeks). control = placebo + SPC, test = probiotic + SPC.

**Figure 3:**
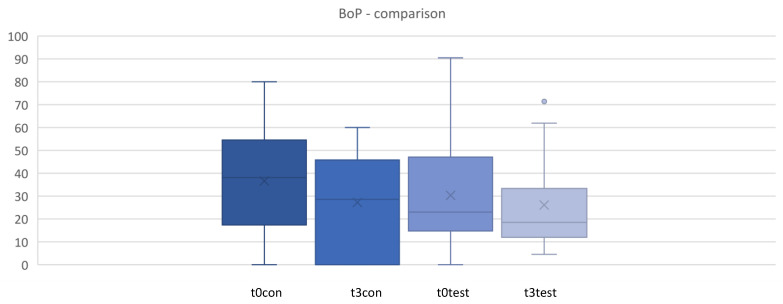
Comparison for bleeding on probing (BoP) between t0 and t3. t0con = t0 control/t3con = t3 control (12 weeks)/t0test = t0 test/t3test = t3 test (12 weeks). control = placebo + SPC, test = probiotic + SPC.

A reduction in bleeding on probing (BoP) was demonstrated in both groups (Figure 2). In the test group, the decrease is most pronounced after 4 weeks and remains at a lower level in the test and control groups over the entire period. The dispersion of the values was lower in the test group than in the control group at all measured time points. The differences in BoP values are statistically significant between the two treatment groups (*p* < 0.05) (Figure 2 and Figure 3).

The results show a statistically significant reduction in bleeding on probing, while all other parameters show no significant changes. Despite comparable conditions between the two groups (PPD, CAL, PCR, and BoP), the probiotic seems to have a positive influence on the gingival inflammatory response.

### 3.2. Plaque Control Record (PCR)

**Figure 4:**
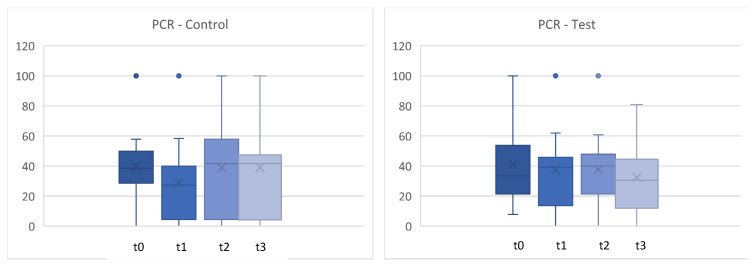
Plaque control record (PCR) in % between t0 and t3. t0 con = t0 control/t3con = t3 control (12 weeks)/t0 test = t0 test/t3test = t3 test (12 weeks). control = placebo + SPC, test = probiotic + SPC.

**Figure 5:**
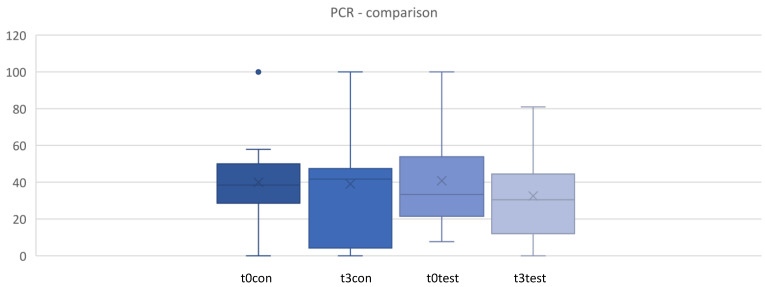
Comparison for plaque control record (PCR) in % between t0 and t3. t0con = t0 control/t3con = t3 control (12 weeks)/t0 test = t0 test/t3 test = t3 test (12 weeks). control = placebo + SPC, test = probiotic + SPC.

Figure 4 shows the percentage of sites with plaque during the clinical trial. No statistically significant differences were found in the change in PCR either (*p* > 0.05). (Figure 4 and Figure 5).

### 3.3. Probing Pocket Depth (PPD)

**Figure 6:**
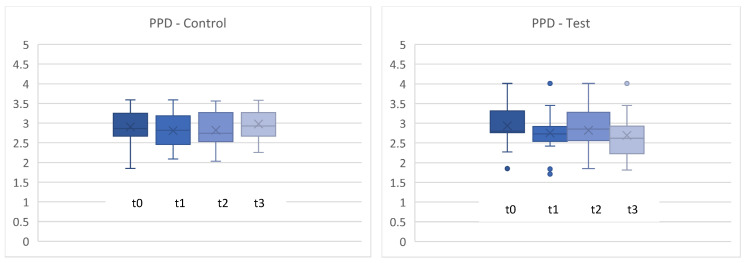
Periodontal pocket depth (PPD) in mm for t0, t1 (4 weeks), t2 (8 weeks), t3 (12 weeks). control = placebo + SPC, test = Probiotic + SPC.

**Figure 7:**
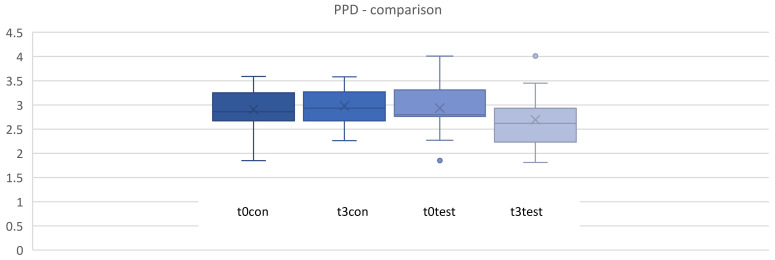
Comparison for periodontal pocket depth (PPD) in mm between t0 and t3. t0con = t0 control/t3con = t3 control (12 weeks)/t0test = t0 test/t3test = t3 test (12 weeks). control = placebo + SPC, test = probiotic + SPC.

As shown in Figure 6, both patient groups were similar in mean PPD at baseline. The study treatment resulted in small reductions in full-mouth PPD over time. No statistically significant inter-group differences could be observed for full-mouth PPD reduction between baseline (t0) and after 4, 8, and 12 weeks (*p* > 0.05) (Figure 6 and Figure 7). 

### 3.4. Clinical Attachment Level (CAL)

**Figure 8:**
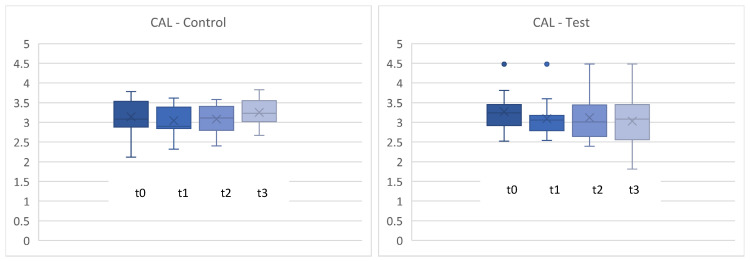
Clinical attachment level (periodontal pocket depth + recession) in mm for t0, t1 (4 weeks), t2 (8 weeks), t3 (12 weeks). control = placebo + SPC, test = probiotic + SPC.

**Figure 9:**
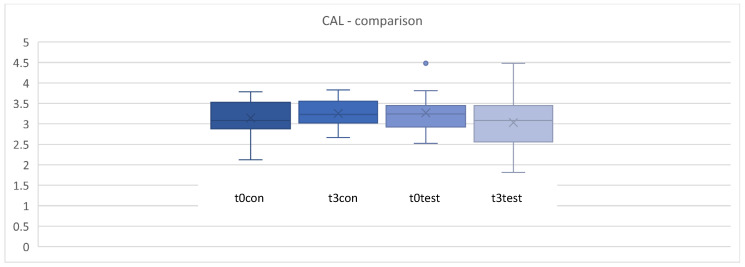
Comparison for clinical attachment level (CAL) in mm between t0 and t3. t0con = t0 control/t3con = t3 control (12 weeks)/t0 test = t0 test/t3 test = t3 test (12 weeks). control = placebo + SPC, test = probiotic + SPC.

The clinical attachment level (CAL) is composed of the sum of the PPD and the extent of gingival recession. Therefore, changes in CAL depend more on a reduction in PPD than on a change in recession. As shown in Figure 8 and Figure 9, no statistically significant differences were found between the test and control groups at baseline and after 4, 8, and 12 weeks for CAL (*p* > 0.05) (Figure 8 and Figure 9).

### 3.5. Individual Patient Analysis

**Figure 10:**
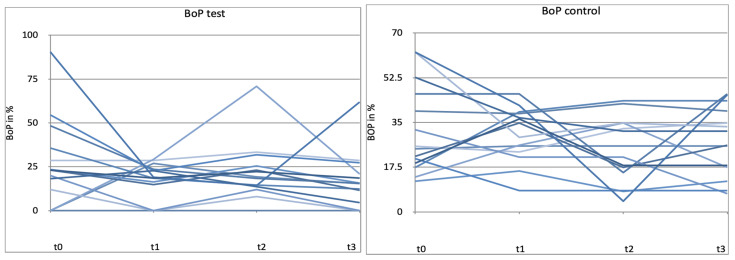
Patient-related diagram for bleeding on probing (BoP) in % for t0, t1 (4 weeks), t2 (8 weeks), t3 (12 weeks) control = placebo + SPC, test = probiotic + SPC. Every line represents one patient.

**Figure 11:**
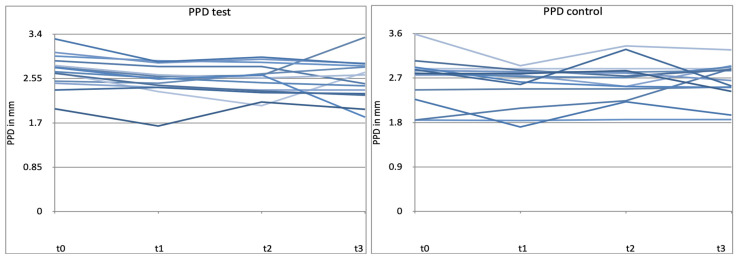
Patient-related diagram for periodontal pocket depth (PPD) in mm for t0, t1 (4 weeks), t2 (8 weeks), t3 (12 weeks). control = placebo + SPC, test = probiotic + SPC. Every line represents one patient.

**Figure 12:**
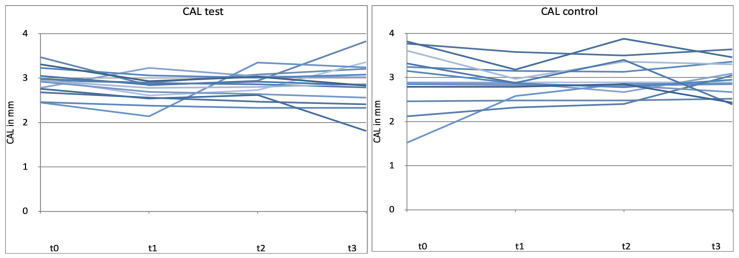
Patient-related diagram for clinical attachment level (CAL) in mm for t0, t1 (4 weeks), t2 (8 weeks), t3 (12 weeks) control = placebo + SPC, test = probiotic + SPC. Every line represents one patient.

As no statistically relevant differences could be detected between the test and control groups, it was decided to investigate and analyze the effect of probiotics on individual patients. The two groups (test and control) were compared. Patient-specific progressions are clearly recognizable (Figure 10, Figure 11 and Figure 12). The test group showed a lower dispersion of parameter values at patient level compared to the control group from time t1. The more homogeneous course of the parameters for BOP, PPD, and CAL for the period t1–t3 indicates a positive influence of the probiotic.

**Figure 13:**
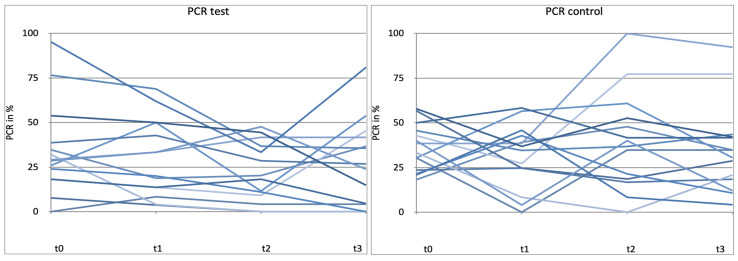
Patient-related diagram for plaque control record (PCR) in % for t0, t1 (4 weeks), t2 (8 weeks), t3 (12 weeks). control = placebo + SPC, test = probiotic + SPC. Every line represents one patient.

We were unable to detect any differences between the two groups for PCR (Figure 13). In addition, values for PCR are very inhomogeneous. The PCR parameter is strongly dependent on the individual oral hygiene of the patients and is apparently least influenced by the intake of *L. reuteri*.

## 4. Discussion

To our best knowledge, this is the first double-blind, placebo-controlled RCT to investigate the effects of *L. reuteri*-containing lozenges on clinical parameters given adjunct once a day for 3 months in supportive periodontal therapy.

PPD, CAL, and PCR showed no statistically significant differences between the test and placebo groups. BoP, on the other hand, was statistically significantly reduced in the test compared to the placebo group. Probably no changes could be detected with regard to residual pockets, CAL, and PCR, as our study group is a very well-supported patient group in supportive periodontal therapy. Furthermore, a possible underpowering could be another reason.

To the authors’ best knowledge, there has only been one study to date to investigate *L. reuteri*-containing lozenges in supportive treatment of periodontitis. Grusovin et al. presented 1-year results of the clinical efficacy of *L. reuteri*-containing lozenges in the supportive therapy of generalized periodontitis stages III and IV [14]. The results after one year were more impressive than the results of this study. The test group showed a statistically significant deeper reduction in PPD (all time points), an increased BoP reduction (6 and 9 months), and a higher PAL (probing attachment level) gain (6 months). The better effects might be related to the longer follow-up period (3, 6, 9, and 12 months). Moreover, the *Lactobacillus*-containing lozenge was given twice a day for 3 months and not only once a day. It was to be expected that with a minimum dose of one tablet over 3 months and a follow-up period of only 4, 8, and 12 weeks, no comparable results could be described; rather, the aim was to test whether clinically and statistically significant effects could be detected at all.

Furthermore, the number of cases could be a reason for the poorer results of the present study. Perhaps because of the small number of patients, only small differences could be detected between the groups. However, the number of cases in the study mentioned above was even smaller (20 in total). In principle, further studies with higher case numbers are necessary to verify the results demonstrated.

The discussion of the presented results is difficult. As already mentioned, sufficient studies are lacking to classify the outcomes in the current literature. So far, more research has been done on the effect of (adjunctive) *L. reuteri* on the clinical and microbiological outcome of patients with periodontitis undergoing non-surgical periodontal treatment for the first time [2,4]. Long-term follow-up, a larger number of patients, and the heterogeneity of studies were often criticized in this context [15].

To the best of the authors knowledge, this is the first study to analyze individual patient outcome after the administration of probiotics. Interestingly, patients have responded very differently to the therapy in the test group, also in the control group. This explains why probiotic therapy in its current form is controversial, as no extensive effects on patient cohorts can be determined [9]. Possibly, the individual reaction of patients to the probiotic therapy is due to the fact that the oral microbiome is very individual and even differs in the oral cavity of an individual patient [16]. Furthermore, other factors influencing the periodontal state, like the individual immune response and other environmental and systemic risk factors, must be taken into consideration [17]. No effects on the plaque control record were found in any group. This is understandable, as the plaque control record depends primarily on daily oral hygiene.

Since sufficient studies are missing, it is not yet scientifically precisely defined how long *L. reuteri*-containing lozenges should be administered and at what dose in supportive periodontal therapy. Our study shows, for the first time, that the first clinical effects (reduction of BoP) can already be achieved after only 3 months, starting in the first 4 weeks, with only one lozenge per day. A minimum application period of 3 months was selected according to the application protocol for the product used in this study. Since the application protocol does not provide any guidelines for the use of probiotics in follow-up care due to a lack of scientific data as described above, the protocol was based on guidelines for the severity of periodontitis, for which the application protocol is providing information. This still refers to the old classification, explaining application in initial, moderate, and severe. In supportive periodontal therapy, patients usually have moderate periodontitis. For a minimum dose of one tablet a day, an application period of at least 4 weeks, in principle, 12 weeks, is recommended [18].

It could not be verified whether the patients have really taken the lozenges regularly, even if the number of lozenges used was monitored. Furthermore, it is not known whether the lozenges were merely swallowed or actually sucked. It was not possible to control the way the tablets were taken since the procedure took place at home.

As there is a minimal risk of probiotics themselves causing diseases, clinical-side effects must be recorded [19]. In the present study, no adverse health effects were mentioned by the patients. The 28 patients ultimately included were very compliant. Unfortunately, eight patients had to be excluded due to several reasons. Firstly, some had been absent for a longer period, no longer being able to attend the follow-up appointments. Secondly, only healthy men and women were to be included in the study, and patients no longer consistently fulfilling these criteria had to be excluded. Thirdly, post-COVID-syndrome patients no longer fulfilled the criteria of being healthy. By definition, physical and neuropsychiatric symptoms persist for more than 12 weeks without an alternative explanation [20].

Another limitation of this study is the fact that no microbiological studies were performed. The extent to which *L. reuteri* colonized the oral cavity and the oral microbiome changed as a consequence was not investigated. However, microbiological investigations were not the aim, but much more clinical aspects. 

It is difficult to compare probiotics in periodontal supportive therapy with other adjunctive measures because, to the authors’ knowledge, there is no literature drawing a direct comparison to date. A review by Calciolari et al. investigated the efficacy of adjunctive periodontal therapies (also probiotics) in supportive periodontal therapy in patients with residual pockets. They found out there is too little evidence to date to determine the efficacy of an adjunctive strategy in the treatment of residual pockets [21]. However, to the best of the authors’ knowledge, no study was found that made a direct comparison between supplemental probiotic therapy and other adjunctive therapy interventions.

In summary, for some patients, the present study was able to show clinical effects in periodontal supportive therapy with the administration of one *L. reuteri*-containing lozenge (DSM17938 and ATCC PTA5289) per day already after 3 months. However, other patients did not respond to the therapy. It must be pointed out that this statement cannot be transferred to other probiotics or other *L. reuteri* strains being used, since strains can act differently, sometimes in opposite ways. Equating strain effects with species effects is therefore difficult. Microbiological diagnostics and analysis were not performed in this study. The microbiological effects of *L. reuteri* in supportive therapy are not described yet, although some research on the microbiological effects of probiotics as an adjunctive treatment to periodontal therapy in general is available, including *L. reuteri* [6,22]. Therefore, further studies should aim to investigate the oral microbiome when applying *L. reuteri* (DSM17938 and ATCC PTA5289), e.g., via metagenomics and, furthermore, metabolomics. Moreover, the demonstrated clinical results should be further verified by studies with *L. reuteri* in supportive periodontal therapy with a higher number of cases.

## 5. Conclusions

In some patients, the oral usage of one *L. reuteri* lozenge per day for 3 months improved clinical parameters in supportive periodontal therapy. However, this was not the case for all of the included patients. An individual response to therapy with *L. reuteri* must be recorded. Further investigations are needed, especially with larger study groups, to increase the significance of the reported results. Furthermore, investigations of the oral microbiome and possible changes during the intake of the lozenges should be conducted with an adequate microbiological method like metagenomics in a follow-up study.

## Figures and Tables

**Table 1:** Patient demographics.

	Probiotic + SPC	Placebo + SPC
Patients	14	14
Female	10	8
Male	4	6
Age	57.07 ± 3.65	54.75 ± 7.43

## Data Availability

The data supporting the findings of this study are available from the corresponding author upon reasonable request.
